# Detection of Risky Driving Behaviors in the Naturalistic Environment in Healthy Older Adults and Mild Alzheimer’s Disease

**DOI:** 10.3390/geriatrics3020013

**Published:** 2018-03-21

**Authors:** Jennifer D. Davis, Shuhang Wang, Elena K. Festa, Gang Luo, Mojtaba Moharrer, Justine Bernier, Brian R. Ott

**Affiliations:** 1 Department of Psychiatry & Human Behavior, Alpert Medical School of Brown University, Providence, RI 02903, USA; 2 Schepens Eye Research Institute, Mass Eye and Ear, Harvard Medical School, Boston, MA 02114, USA; shuhang_wang@meei.harvard.edu (S.W.); gang_luo@meei.harvard.edu (G.L.); mojtaba_moharrer@meei.harvard.edu (M.M.); 3 Department of Cognitive, Linguistic and Psychological Sciences, Brown University, Providence, RI 02912, USA; elena_festa@brown.edu; 4 Alzheimer’s Disease and Memory Disorders Center, Rhode Island Hospital, Providence, RI 02903, USA; justine.bernier@lifespan.org (J.B.); brian_ott@brown.edu (B.R.O.); 5 Department of Neurology, Alpert Medical School of Brown University, Providence, RI 02903, USA

**Keywords:** Alzheimer’s disease, cognitive impairment, naturalistic driving assessment

## Abstract

Analyzing naturalistic driving behavior recorded with in-car cameras is an ecologically valid method for measuring driving errors, but it is time intensive and not easily applied on a large scale. This study validated a semi-automated, computerized method using archival naturalistic driving data collected for drivers with mild Alzheimer’s disease (AD; *n* = 44) and age-matched healthy controls (HC; *n* = 16). The computerized method flagged driving situations where safety concerns are most likely to occur (i.e., rapid stops, lane deviations, turns, and intersections). These driving epochs were manually reviewed and rated for error type and severity, if present. Ratings were made with a standardized scoring system adapted from DriveCam^®^. The top eight error types were applied as features to train a logistic model tree classifier to predict diagnostic group. The sensitivity and specificity were compared among the event-based method, on-road test, and composite ratings of two weeks of recorded driving. The logistic model derived from the event-based method had the best overall accuracy (91.7%) and sensitivity (97.7%) and high specificity (75.0%) compared to the other methods. Review of driving situations where risk is highest appears to be a sensitive data reduction method for detecting cognitive impairment associated driving behaviors and may be a more cost-effective method for analyzing large volumes of naturalistic data.

## 1. Introduction

Driving is one of the most complex instrumental activities of daily living that the majority of older adults engage in on a regular basis. There are more than 29 million drivers over the age of 65 in the United States, which is 14.6% of the driving population. The United States Department of Transportation estimates that the total miles driven by individuals 65 and older will increase by 50 percent by 2020. Based on overall crash occurrences per year, older drivers have fewer crashes than younger drivers, largely due to reduction in driving miles. When gauged on a per mile basis, however, older drivers are a significant risk group, with crash rates approaching those seen in teenage drivers [1]. Moreover, due to frailty, they are more likely to die in a motor vehicle accident than younger drivers [1,2].

Older drivers with dementia are at a particularly high risk for unsafe driving, carrying a relative crash risk of 2–5 times that seen in comparable healthy elders [3]. Functional brain imaging studies suggest that disruption of visual processing circuits of the brain linking the occipital and prefrontal regions, particularly in the right hemisphere, leads to increasing degrees of driving impairment over time that ultimately precludes safe driving in all persons with Alzheimer’s disease (AD) [4,5]. Currently, it is estimated that there are over 5 million people in the United States with Alzheimer’s disease (AD), and the prevalence will increase to epidemic proportions as our population ages in the absence of an effective prevention strategy. Most will continue to drive, at least in the early stage of their illness, despite their cognitive impairment.

Past research studies on cognitively impaired drivers have employed road tests and simulators as proxy measures of driving performance or epidemiologic outcomes such as crashes as measures of driving safety. These studies provide valuable insights into the potential problems posed by this group of at-risk drivers, but each method has limitations. The low frequency of crash rates in this population limits epidemiologic outcomes to retrospective studies or prospective studies spanning years and involving hundreds of participants in order to include enough events. Furthermore, crashes may be due to environmental conditions and the fault of other drivers [6]. Caregiver reports have limited predictive value, possibly due to personal bias [7,8]. On-road tests are only snapshots of real-world driving and may be affected by psychological factors, such as anxiety [9] and best behavior, as well as environmental factors [10]. Importantly, road tests only measure driving performance rather than driving safety, which is known to be strongly affected by driving habits, including restricted driving areas and mileage. Although simulator studies can investigate driving behavior in unexpected and possibly unsafe situations, they are only proxy measures of driving that are limited by similar factors as road tests, as well as by simulator sickness [11].

Naturalistic studies, a relatively new and novel approach, are appealing because of their face validity but are difficult to carry out due to the large volumes of data collected [12,13]. In a previous study, we compared road test performance to four hours of home driving videos for each participant. We found a modest relationship between driving performance in the two settings, and only the road test weakly correlated with crashes occurring in the three years prior to enrollment [14]. In that study, one potential reason for the relatively weak correlation between naturalistic driving and road test performance was sampling error, since only four hours of naturalistic driving was analyzed from the two weeks collected (less than 50%) due to the challenges of quantifying large volumes of naturalistic data. This is not atypical for naturalistic research. For example, the 100-car project collected data from 100 volunteers across a one-year period resulting in 42,300 h of driving data [15]. To manually review this volume of data is practically impossible and fully automated analyses of such big data are still in development.

Given that naturalistic data analysis is informative but limited by the lack of available methods to analyze large volumes of data, the goal of this study was to apply a semi-automated data reduction method to archival naturalistic driving videos reported on previously (see [14]). Goals of the study were to identify potential unsafe behaviors in four key driving situations that are likely to have high crash risk and/or indicate distraction (i.e., rapid stops, lane deviations, turns, and intersections). Rigorous validation tests [16] against manually labeled results showed a sensitivity of informative epoch detection ranging from 85% to 100% and false detection rate under 5%. In addition, the validity of this data reduction method was investigated by relating error scores generated by the computerized method to error scores on a road test and cognitive measures of executive functioning. It was hypothesized that older adults with cognitive impairment due to early AD would make more errors and more severe errors in these situations compared to healthy older adults and that these scores would be related to independent on-road driving test scores. Secondary goals were to identify which types of errors best predicted group membership to better understand cognitive impairment associated driving behaviors.

## 2. Materials and Method

### 2.1. Participants

Study participants were 44 older adults diagnosed with Alzheimer disease (AD) and 16 older adults with normal cognition served as healthy controls (HC). Demographic characteristics are presented in Table 1. Participants for whom GPS data were available were drawn from a larger study of naturalistic driving for which the method is described in detail elsewhere [14]. All participants had a valid driver’s license and no at-fault crashes within one year prior to enrollment. Participants were recruited primarily from a hospital-based memory disorders clinic during routine follow-up visits. Disease severity was measured with the Clinical Dementia Rating scale (CDR) [17]. Healthy participants were required to be a CDR = 0 indicating no cognitive impairment; only patients very mild or mild cognitive changes as indicated by CDR scores of 0.5 or 1 were included in the cognitively impaired group. Cognitively impaired participants met diagnostic criteria for possible or probable Alzheimer’s disease (AD) based on NINCDS-ADRDA criteria [18]. Patients were on a stable dose of a cholinesterase inhibitor for six weeks, if prescribed. Exclusion criteria included reversible causes of cognitive impairment, physical or ophthalmologic disorders that might impair driving abilities, intellectual disability, schizophrenia, bipolar disorder, or alcohol/substance abuse within the previous year. Anxiolytic and antipsychotic medications were permitted, but dosages were required to be stable for six weeks before study entry. Written informed consent was obtained from all participants before they participated in the study. The study was conducted in accordance with the Declaration of Helsinki, and the protocol was approved by the Institutional Review Board at Rhode Island Hospital (Project #001207; parent study approved on 2 January 2007 and secondary analyses approved on 12/3/2014). As described below, all participants were required to pass a standardized road test before being allowed to have cameras installed in the car for monitoring. This is considered the gold standard method for assessing driving safety in older drivers.

Participants were selected for inclusion in the current study for secondary analysis if they had GPS data and at least one hour of video for analysis. From the total sample of 65 participants with cognitive impairment, 44 had data sufficient for automated analyses and 16 out of 31 healthy controls were also analyzed with this method to serve as a comparison group.

### 2.2. Procedure

#### 2.2.1. Standardized Road Test

Participants were administered the Rhode Island Road Test (RIRT) by a professional driving instructor with extensive experience working with older adults with neurological diseases and cognitive impairment. The instructor was blind to group membership. The RIRT shows adequate inter-rater reliability [14]. The RIRT was administered within one month of the baseline office assessment during daylight hours under good road conditions. The test covered 6.5 miles of urban terrain without highway driving and required 45 min to complete. The driving instructor accompanied the participant in a specially fitted vehicle that had a brake on the passenger side for emergency use. The driving instructor only provided verbal instructions to complete the course.

Twenty-eight driving maneuvers/behaviors were rated on a three-point scale (0 = unimpaired; 1 = mildly impaired; 2 = moderately to severely impaired) (see [19] for complete description). The majority of participants had the same number of opportunities to engage in a driving maneuver across the test. If an event was not encountered (e.g., response to pedestrian), it was not included in the overall proportional score. Total scores ranged from 0 to 960, with higher scores reflecting poorer performance. The sum of ratings (range 0–2) for each event divided by the number of observed maneuvers served as the outcome variable for statistical analyses.

#### 2.2.2. Naturalistic Driving Recordings

All participants completed an on-road test with a driving instructor. For those participants who passed the road test, continuous video recording was collected over a two-week period. Cameras were low profile and installed on the dash and rear deck, and GPS was monitored by an extension attached to the vehicle roof. Cameras captured front, side, and rear views. Video from the cameras was automatically downloaded to a portable digital video recording device that was placed under the passenger seat. Cameras were installed in the participant’s cars on the same day that they completed and passed the RIRT. Participants were instructed to drive in their typical environment and routine.

Naturalistic driving was rated by a professional driving instructor with the Composite Driving Assessment Scale (CDAS) (see [14] for scale development). Behaviors were divided into discrete events (i.e., maneuvers) and global events (i.e., attention, attitude, reaction time). Because the number and types of driving situations in naturalistic driving assessment cannot be controlled in the same fashion as on a road test, each item was given a global rating of ‘unimpaired’, ‘mildly impaired’, or ‘moderately to severely impaired’ according to the same three-point Likert scale as the RIRT (range 0–2). Total scores ranged from 0 to 60 with higher scores reflecting poorer performance. The error severity score was an average score reflecting the sum of the ratings for each behavior divided by the maximum number of observed behaviors.

#### 2.2.3. Automated Computerized Analysis

Computerized analyses were applied to the naturalistic driving data to identify risky driving behaviors in circumstances or sections, where risk of crash is high (i.e., turns, intersections, rapid stops, and lane deviations). The goal of this approach was to determine if identifying situations in which the risk for driving errors was high could be as informative as viewing continuous video. The used driving data included the front view video, the GPS, and the speed. The GPS and the speed were recorded on the video by DVRs and were recognized from each frame of the video using Optical Character Recognition technique.

Turns: The driving route was divided into segments, each of which was at least 15 m long. Turns were detected by computing the direction change between a segment and the next nonadjacent segment [16]. If the absolute direction change was between 40° and 150°, the segment was labeled as a turn.

Intersections: A database of 654,491 intersections in the New England area was used. The database includes geo-location and the road classes (e.g., highway, major road, local street, etc.) regarding each intersection. An intersection was grouped as a major or minor one, depending on whether one of the intersecting roads is a major road. An intersection event is marked if the car is within 15 m from an intersection.

Rapid Stop: The speed change was calculated based on the speed recorded on the video. For each subject, all the events with speed drop more than 6 mph within 3 s were first identified, and the events were then sorted. A rapid-stop event was flagged if the deceleration was in the top 5% of the events when driving speed higher was faster than 30 mph, or the deceleration was in the top 50% of the events when driving speed was below than 30 mph. The detection criteria were selected after comparing the automated method to full manual review of a subset of driving videos.

Lane Change: The lane marker was determined using a matched filter, and the lane change was detected by tracking the lane marker in the recorded videos. A lane-change event was flagged if a lane marker ran across the middle line of the front view, and the lane-marker lateral shift was greater than half the lane width.

Multi-type: If a driving segment satisfied one or more of the conditions described above, it was labeled as an informative behavior: (1) turned on a road with priority higher than local roads; (2) turned left at an intersection; (3) straight through a major intersection; (4) a rapid-stop event happened; (5) a lane-change event happened. Events could be a single type or multi-type involving combinations of event types.

#### 2.2.4. Quantification of Detected Events

Events flagged by the computerized method were manually reviewed by five staff. Ratings were agreed upon by consensus. Each event was rated according to a Modified Mockingbird Event Scoring System. The original scoring system was developed by a commercial driving evaluation system, DriveCam^®^. This method has been previously used to characterize and monitor driving in a longitudinal study of ambulance drivers [20]. Unsafe driving events were rated according to eight major categories of concern: distractions, poor awareness, driver conduct, fundamentals, following too close, driver condition, traffic violations, and other concerns. The specific events or problems were graded for safety risk on a 0–10 point demerit scale. A single unsafe driving event could have more than one demerit category, such as judgment error combined with poor awareness of intersection, leading to a combined driving severity rating score for the individual items. Mockingbird scores were corrected for self-reported mileage. Near collisions and collisions were also recorded.

#### 2.2.5. Cognitive Measures

In addition to the Mini Mental State Exam, a measure of global cognition [21], several cognitive measures psychomotor speed and executive functioning were administered to evaluate the validity of the automatized method of event detection. Measures were selected based on their relationship to driving behavior [22]. A trained research assistant administered the clinical measures at the baseline office visit. Raw scores were used in data analyses.

Trail Making Test, Parts A & B [23]: Trail Making, Part A is a test of visual search and psychomotor speed. In Part A, the subject connects a series of encircled numbers in order. In Part B, the participant connects a series of encircled numbers and letters in order, alternating between number and letter. Part B is considered an executive functioning task as it requires more complex attention and set shifting. Performance is measured in time to complete the test (maximum time set at 180 s for A and 300 s for B). Time to completion served as the dependent variable in analyses.

Maze Drawing: Two different maze drawing tasks were administered, one by paper and pencil and the other by computer. Maze drawing assesses multiple cognition functions and has been postulated to serve as a proxy measure for basic street navigational skills [24,25,26]. Time to completion and errors (i.e., dead-end errors) served as the dependent variables in analyses.

Clock Drawing: Participants were given a blank piece of paper and instructed to draw a clock, put all of the numbers in and set the time to 10 after 11. Clocks were scored using Freund’s seven-point scoring system [27].

#### 2.2.6. Miles Driven and Crash History

At the baseline study visit, miles driven per week and crash history were obtained. A study partner who drives with the participant at least once per week provided that information for the cognitively impaired participants.

#### 2.2.7. Statistical Analyses

Independent sample *t*-tests compared group differences on demographic characteristics. Pearson correlations between each driving measure (54 possible measures, according to Mockingbird scores) and participant group (AD vs. HC) were computed to inform logistic modeling. Rather than using a summed error score, as used in past research, this approach was used in order to take multiple error ratings into consideration given the high number of error types and no a priori prediction for how they should be combined in this population. The top eight measures were applied as features to train a logistic model tree classifier, which was used to predict group membership (AD vs. HC).

## 3. Results

All videos analyzed for this study were recorded during the period between December 2007 and January 2010. No motor vehicle crashes were observed in any of the videos analyzed. Cognitively impaired drivers had an average of 404.47 min of recorded driving (SD = 65.20, range = 50.12–2592.52) while control group drivers had an average of 469.40 min (SD = 386.20, range = 155.9–1747.55). Demographic characteristics are presented in Table 1. As expected, global cognition as measured by the Mini Mental State Exam (MMSE) was lower in AD participants, and AD participants drove fewer miles per day. Overall, however, groups were well matched on gender, education, driving experience, and overall mileage available for analysis.

From all flagged events by automated detection system, 879 events were rated as events with errors during the manual review. These events were manually reviewed and rated with the Mockingbird scoring system. The majority of rated events were rapid stops (42.7%) and multiple events (41.2%) with only 9.9% lane deviations, 4.8% turns, 1.5% intersections. When controlling for mileage driven, AD participants had higher error scores (mean = 0.74, SD = 0.66) than controls (mean = 0.23, SD = 0.14; *t*[58] = −2.98, *p* = 0.001).

Pearson correlations were conducted to determine which types of errors coded with the Mockingbird rating system related best to diagnostic group. Eight Mockingbird driving errors correlated significantly with group membership (AD vs. HC). Among these measures, AD patients made more severe errors for lane maintenance and not looking far enough ahead, while the control group made more severe errors for behaviors related to distraction, failing to keep an out, and generally more risky behaviors (see Table 2).

These eight Mockingbird error types were then applied to train a logistic model tree classifier to predict group membership. The prediction of AD diagnosis with these eight Mockingbird scores resulted in the highest overall accuracy, and the sensitivity and specificity were also considerably high relative to error scores on the road test and continuous review of the naturalistic driving videos (see Table 3). Total overall accuracy score was reduced when total Mockingbird error score was corrected for self-reported miles driven.

One way to examine the validity of the automated method as a viable data reduction method is to relate it to performance on a standardized road test. Total error score, corrected for mileage, was significantly correlated with the road test error score (*r* = 0.30, *p* = 0.02). To further evaluate the validity of the automated method, Mockingbird error score (corrected for miles driven), was correlated with clinic measures of cognitive functioning. In the overall group, there were modest correlations between error scores and global cognition as measured by the MMSE (*r* = −0.34, *p* = 0.01), time to perform mazes (*r* = 0.26, *p* = 0.049), and number of errors on mazes (*r* = 0.27, *p* = 0.04). There was no relationship between error scores and clock drawing (*r* = 0.01, *p* = 0.931), Trails A time (*r* = 0.02, *p* = 0.180), or Trails B time (*r* = 0.13, = *p* 0.308).

## 4. Discussion

This study evaluated the utility of an automated data reduction system for naturalistic driving data to identify discrete driving situations where errors or crashes may be more likely to occur. Overall, AD participants made more egregious errors than cognitive healthy older adults matched on demographic and many driving variables with the exception of mileage; AD drivers drove fewer miles per day, so analyses controlled for miles driven. When specific types of errors were analyzed, AD patients tended to have difficulty with fundamentals of driving, particularly lane maintenance and looking far enough ahead to anticipate traffic situations and maneuvers, such as turns. This is consistent with prior research showing that lane deviation is one of the most common error types made in AD drivers [14,28]. In contrast, cognitively healthy drivers tended to make errors when they were multi-tasking and potentially distracted by using a phone or car technology (e.g., radio, eating or passenger conversations). HCs were also more likely to engage in these as well as other risky behaviors, such as pulling out too quickly in traffic or not leaving room for potential exit in tight traffic situations (i.e., ‘leaving an out’).

When compared to error ratings of a standardized road test and error ratings of a composite rating of naturalistic driving, the Mockingbird scoring of discrete events achieved the highest sensitivity and specificity in predicting AD diagnosis. As such, this method of detailed analysis of potentially high risk driving situations within naturalistic data may be more informative in clinical applications than composite ratings.

It is of interest that although AD patients overall made more driving errors, control group drivers showed more risky driving behavior, particularly related to engaging in distracting behaviors. It is possible that cognitively impaired drivers, self-control their exposure to risky driving situations and engage less in distracting behaviors to maintain safety. This finding shows the importance of distinguishing driving behavior and driving skill/ability when studying older drivers.

The automated, event-based method appears to be a valid method for data reduction, and it is more clinically informative when examining multiple relevant behaviors simultaneously than relying on global impressions or summed error scores. In-office cognitive measures had significant, but modest, associations with driving errors. This is not surprising as associations between cognition and driving are limited [26,29,30]. Importantly, the automated method showed good overall accuracy for classifying AD diagnosis, suggesting sensitivity to a disease which places individuals at much greater risk for driving safety errors [31].

This study has some limitations. Not all of the sample could be analyzed given lack of GPS data for all participants, risking potential bias in the data. However, given that failure to be included in analyses was related to technical challenges with the camera set-up rather than participant characteristics, it is unlikely that this influenced our findings. Driving behaviors were evaluated with three different scoring methods/scales for the road test, composite assessment of naturalistic driving, and the automated event-based method. As such, there may be differences in the quality of the scoring method that made the automated method the most robust approach to predicting AD diagnosis. Only participants with very mild to mild AD were studied, and it is unclear how this method would apply to individuals with more severe cognitive impairment. Finally, more women comprised the AD group compared to the HC group. The sample size was too small in this study to divide groups by gender to confidently evaluate any potential gender specific differences on driving outcomes. Future research should strive to determine if there are gender differences that emerge in different types of driving situations in relation to cognitive impairment.

Overall, use of a data reduction method to identify driving situations where risk may be greatest appears to be a sensitive tool for detecting cognitive impairment associated with driving behaviors and may be a more practical and cost-effective method for analysis of naturalistic driving data. Future studies may wish to use this method of data reduction to follow AD drivers longitudinally as their cognition declines to better understand the types of driving errors associated with AD progression, to identify when to recommend driving cessation, and to track response to remediation programs.

## Figures and Tables

**Table 1:** Demographic and driving characteristics of participants.

Participant Characteristics	AD (*n* = 44) Mean (SD)	HC (*n* = 16) Mean (SD)
Female (%)	49%	31%
Age (years + SD)	75.11 (6.70)	72.06 (8.35)
Education (years, mean + SD)	13.43 (3.30)	14.56 (2.19)
Mini Mental State Exam (mean + SD max points = 30)	25.56 (2.47) *	29.31 (0.80)
Years driving (mean + SD)	55.44 (9.25)	53.75 (6.96)
Self-reported miles driven per day	15.82 (13.45) *	25.84 (14.48)
Trips taken per day	1.43 (0.91)	2.02 (1.76)
Total driving mileage recorded	187.72 (175.58)	211.41 (197.04)

* *p* < 0.05.

**Table 2:** Correlations between Mockingbird scores and group membership (AD vs. HC).

Mockingbird Error Types	Pearson’s Correlation (*p*-Value)
Unsafe/risky **	0.51 (*p* = 0.03)
Distracted by electronic dDevice **	0.40 (*p* = 0.00)
Unsafe/unnecessary **	0.39 (*p* = 0.02)
Minor lane maintenance error *	0.33 (*p* = 0.04)
Other distraction **	0.31 (*p* = 0.02)
Failed to keep an out **	0.29 (*p* < 0.00)
Distracted by mobile usage **	0.26 (*p* = 0.00)
Not looking far enough ahead *	0.25 (*p* = 0.00)

Note: * indicates significant correlation for AD; ** indicates significant correlation for HC.

**Table 3:** Logistic regression predicting diagnosis (AD vs. HC) from Mockingbird, composite naturalistic ratings (CDAS), and road test ratings (RT).

Driving Error Scores	Overall Accuracy (%)	Sensitivity (%)	Specificity (%)
Mockingbird scores (logistic model tree)	91.7	97.7	75.0
Mean mockingbird scores (corrected for mileage)	76.8	92.7	33.3
HCDAS error rate	60.3	50.0	87.5
RT error rate	72.9	74.4	68.8
